# Association between abdominal aortic calcifications, bone mineral density and vertebral fractures in a cohort of HIV-positive patients

**DOI:** 10.7448/IAS.17.4.19715

**Published:** 2014-11-02

**Authors:** Nathalie Iannotti, Lidia Gazzola, Alessia Savoldi, Elisa Suardi, Viola Cogliandro, Francesca Bai, Alberto Magenta, Mauro Peri, Teresa Bini, Giulia Marchetti, Antonella d'Arminio Monforte

**Affiliations:** 1 San Paolo Hospital, Clinic of Infectious Diseases, Milan, Italy; 2 San Paolo Hospital, Department of Radiology, Milan, Italy

## Abstract

**Introduction:**

Evidence from HIV-negative cohorts suggests a link between osteoporosis and cardiovascular disease. We evaluated the presence and distribution of abdominal aortic calcifications (AAC) and its correlation with bone mineral density (BMD) and vertebral fractures (VF) in a cohort of HIV-positive patients.

**Materials and Methods:**

In this cross-sectional study, 280 asymptomatic HIV-positive patients from the SPID (“San Paolo” Infectious Diseases) cohort were submitted to lateral spine X-ray and DXA. AAC was identified using the AAC-8 score, which estimates the total length of calcification of the anterior and posterior aortic walls in front of vertebrae L1–L4. Low BMD was defined by T-score or Z-score <−1 at lumbar spine or femoral neck. VF were identified by morph-metric analysis of X-ray and were defined by the “spine deformity index” (SDI) ≥1 according to semiquantitative method by Genant. Associations between AAC, BMD and SDI were evaluated by univariate and multivariate logistic regression models. The relationship between the grade of AAC and SDI was evaluated by Spearman's correlation.

**Results:**

AAC≥1 was present in 65 patients (23.2%); of these 15 patients showed moderate/severe calcifications (AAC>2). Low BMD was found in 163 patients (58.2%) and VF (SDI≥1) in 47/274 patients (17.1%). By univariate analysis, factors associated with AAC>=1 were: age (for additional 10 years older HR 3.81 [IC95% 2.64–5.51], p<0.0001) lower CD4 nadir (for additional 50 CD4 HR 0.89 [IC95% 0.82–0.97], p=0.01) AIDS-diagnosis (HR 2.13 [IC95 % 1.11–4.08], p=0.02) and being on HAART (HR 2.75 [IC95% 1.28–5.90], p=0.009). In multivariate analysis, only age (OR 2.62, IC95% 1.72–3.99, p<0.0001) resulted significantly associated with AAC≥1. Patients with AAC≥1 had twofold increase in the risk of low BMD (HR 2.45 [IC95% 1.32–4.45], p=0.004) and VF (SDI>=1: HR 2.17 [IC95% 1.1–4.2], p=0.02) compared to patients without AAC. The grade of AAC was directly correlated with the grade of SDI (rho=0.16; p=0.008): AAC>2 determines a sixfold increase in the risk of VF (HR 6.44 [IC95% 2.21–18.79], p=0.0006). AAC≥1 predict VF independently from BMD, vitamin D status and bone turnover marker (Table 1).

**Conclusions:**

In our HIV population, AAC resulted a strong predictor of both low BMD and VF, irrespective of factors involved in bone formation. The grade of AAC was directly correlated with the grade of VF.

**Table 1:** Predictors of vertebral fracture in our HIV population

Predictors of Vertebral Fracture in our HIV population	AHR of SDI≥1	IC 95%	p
AAC≥1	2.87	1.30–6.31	.008
Low BMD	0.70	0.33–1.51	.37
Increased bone turnover	1.87	0.84–4.75	.12
Vitamin D deficiency (<30)	1.67	0.52–5.38	.38
Increased PTH levels (>65)	0.66	0.28–1.50	.32
